# Supplementary material for: Trophoblast Cell Surface Antigen 2 (TROP2) as a Predictive Bio-Marker for the Therapeutic Efficacy of Sacituzumab Govitecan in Adenocarcinoma of the Esophagus
Source: Cancers (Basel). 2022 Sep 30;14(19):4789. doi: 10.3390/cancers14194789 (PMC9562858; doi:10.3390/cancers14194789)
Supplement: Supplementary file 1 [file cancers-14-04789-s001.zip › cancers-1917352-supplementary.pptx]

## Slide 1
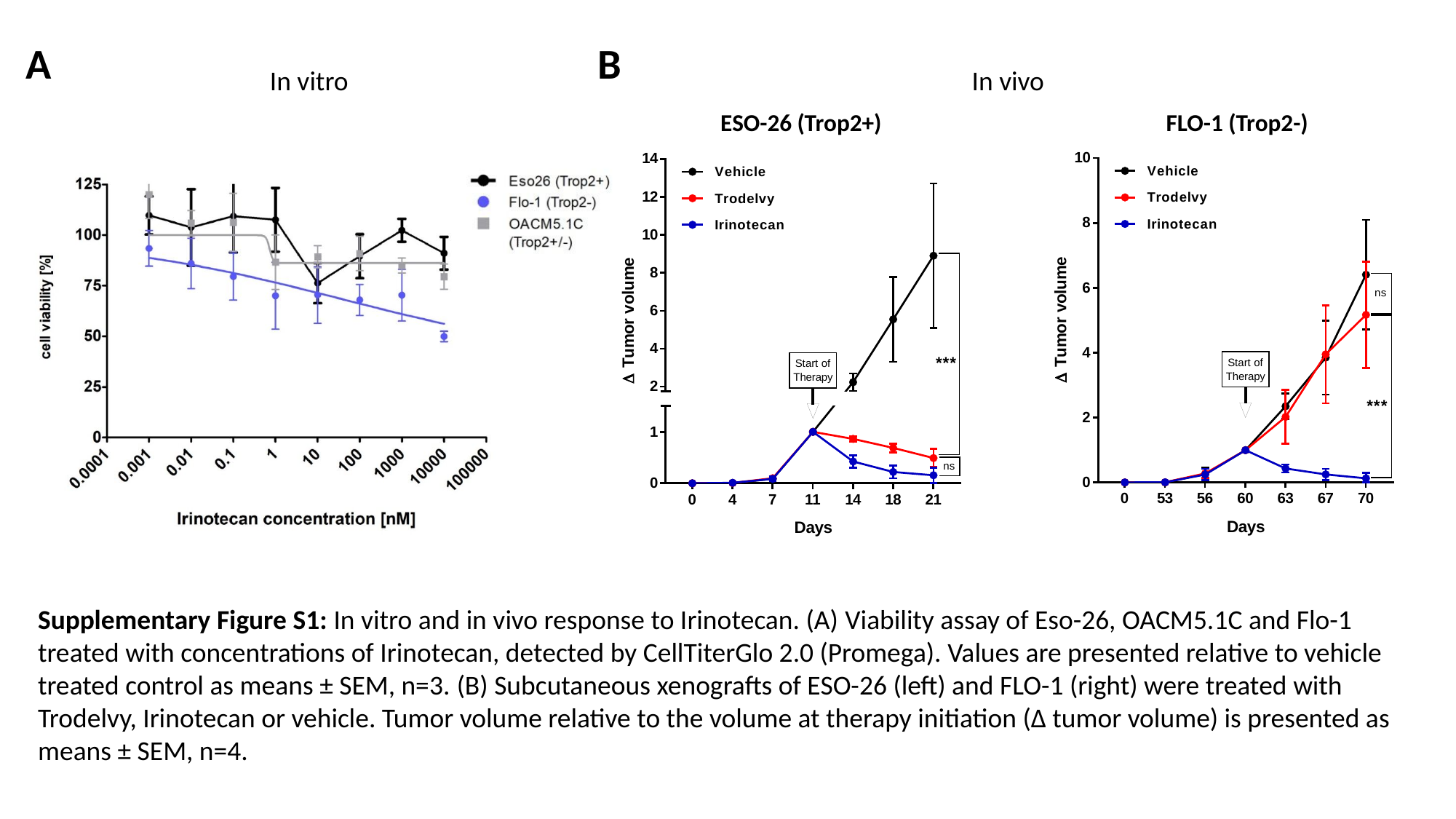

A
B
In vitro
In vivo
ESO-26 (Trop2+)
FLO-1 (Trop2-)
Supplementary Figure S1: In vitro and in vivo response to Irinotecan. (A) Viability assay of Eso-26, OACM5.1C and Flo-1 treated with concentrations of Irinotecan, detected by CellTiterGlo 2.0 (Promega). Values are presented relative to vehicle treated control as means ± SEM, n=3. (B) Subcutaneous xenografts of ESO-26 (left) and FLO-1 (right) were treated with Trodelvy, Irinotecan or vehicle. Tumor volume relative to the volume at therapy initiation (∆ tumor volume) is presented as means ± SEM, n=4.

## Slide 2
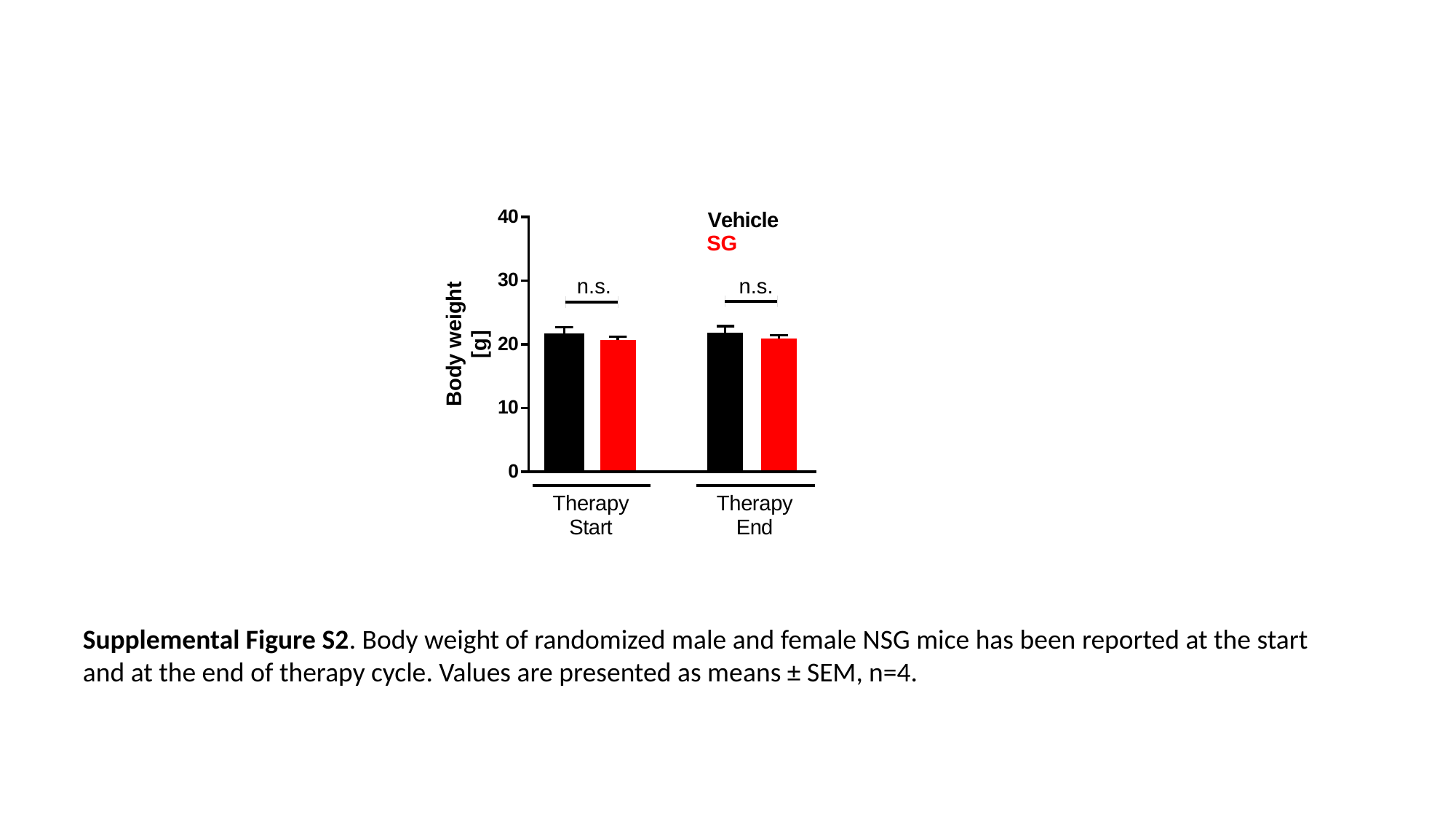

Supplemental Figure S2. Body weight of randomized male and female NSG mice has been reported at the start
and at the end of therapy cycle. Values are presented as means ± SEM, n=4.

## Slide 3
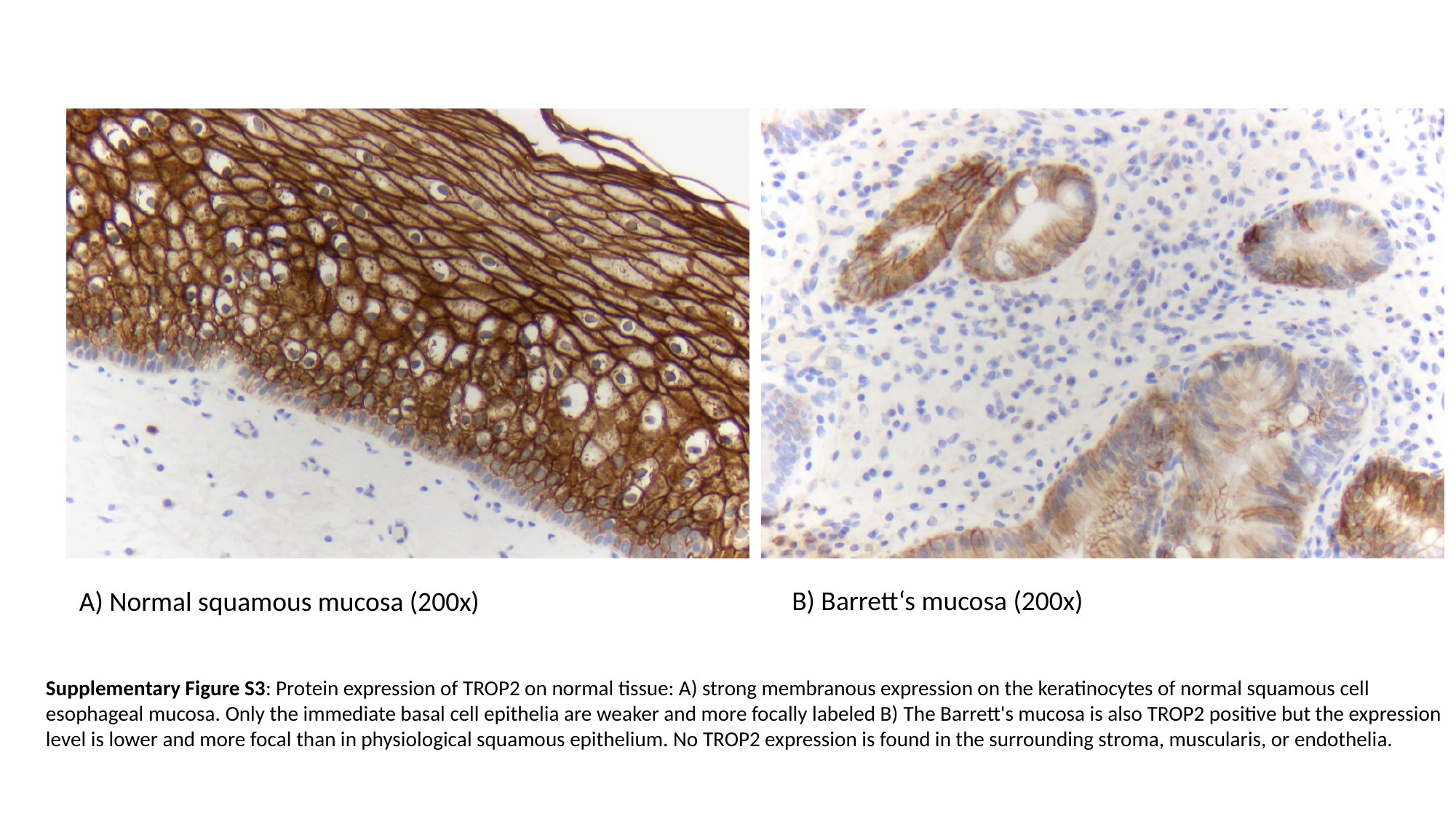

B) Barrett‘s mucosa (200x)
A) Normal squamous mucosa (200x)
Supplementary Figure S3: Protein expression of TROP2 on normal tissue: A) strong membranous expression on the keratinocytes of normal squamous cell
esophageal mucosa. Only the immediate basal cell epithelia are weaker and more focally labeled B) The Barrett's mucosa is also TROP2 positive but the expression
level is lower and more focal than in physiological squamous epithelium. No TROP2 expression is found in the surrounding stroma, muscularis, or endothelia.

## Slide 4
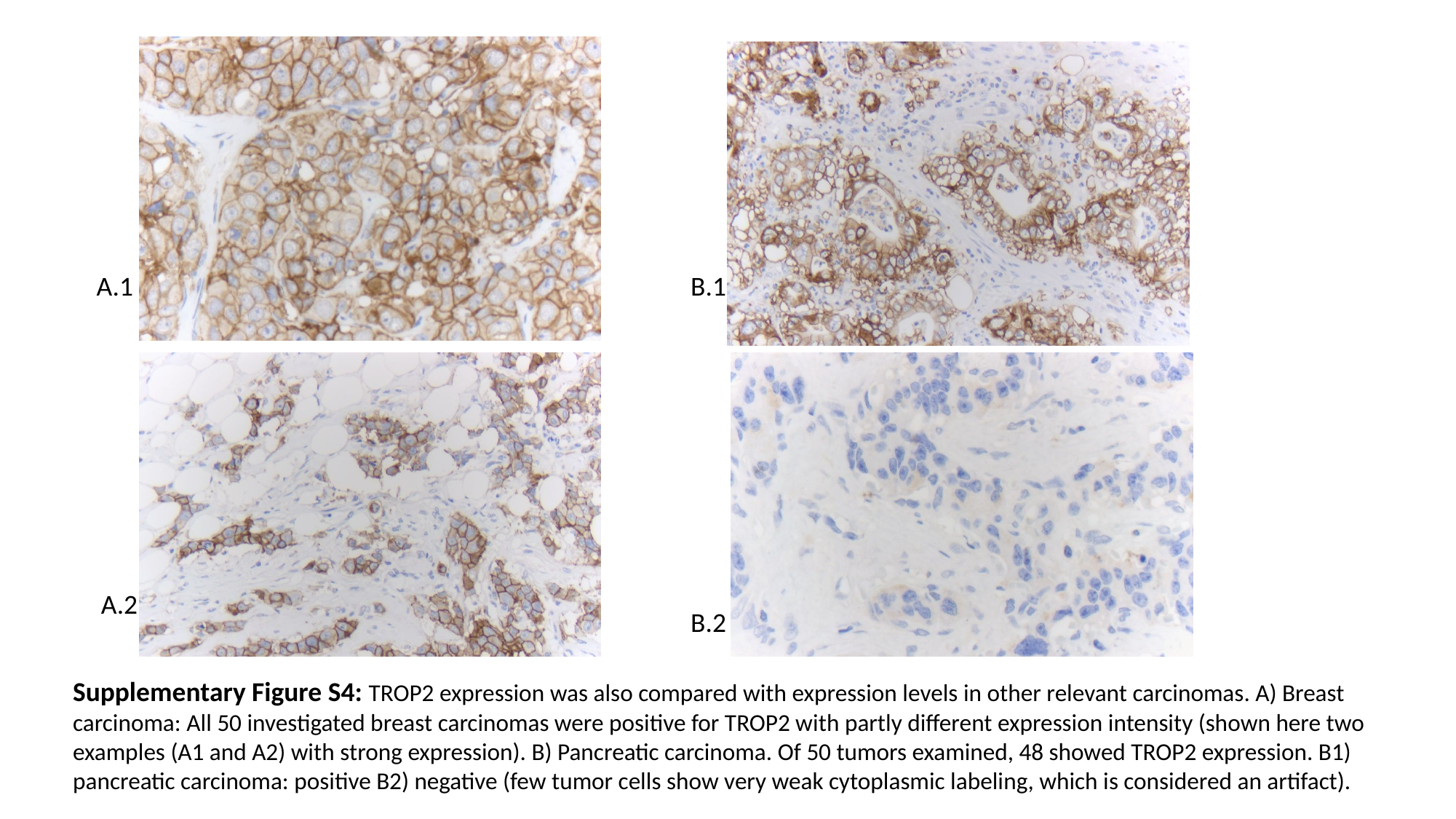

A.1
B.1
 A.2
B.2
Supplementary Figure S4: TROP2 expression was also compared with expression levels in other relevant carcinomas. A) Breast carcinoma: All 50 investigated breast carcinomas were positive for TROP2 with partly different expression intensity (shown here two examples (A1 and A2) with strong expression). B) Pancreatic carcinoma. Of 50 tumors examined, 48 showed TROP2 expression. B1) pancreatic carcinoma: positive B2) negative (few tumor cells show very weak cytoplasmic labeling, which is considered an artifact).

## Slide 5
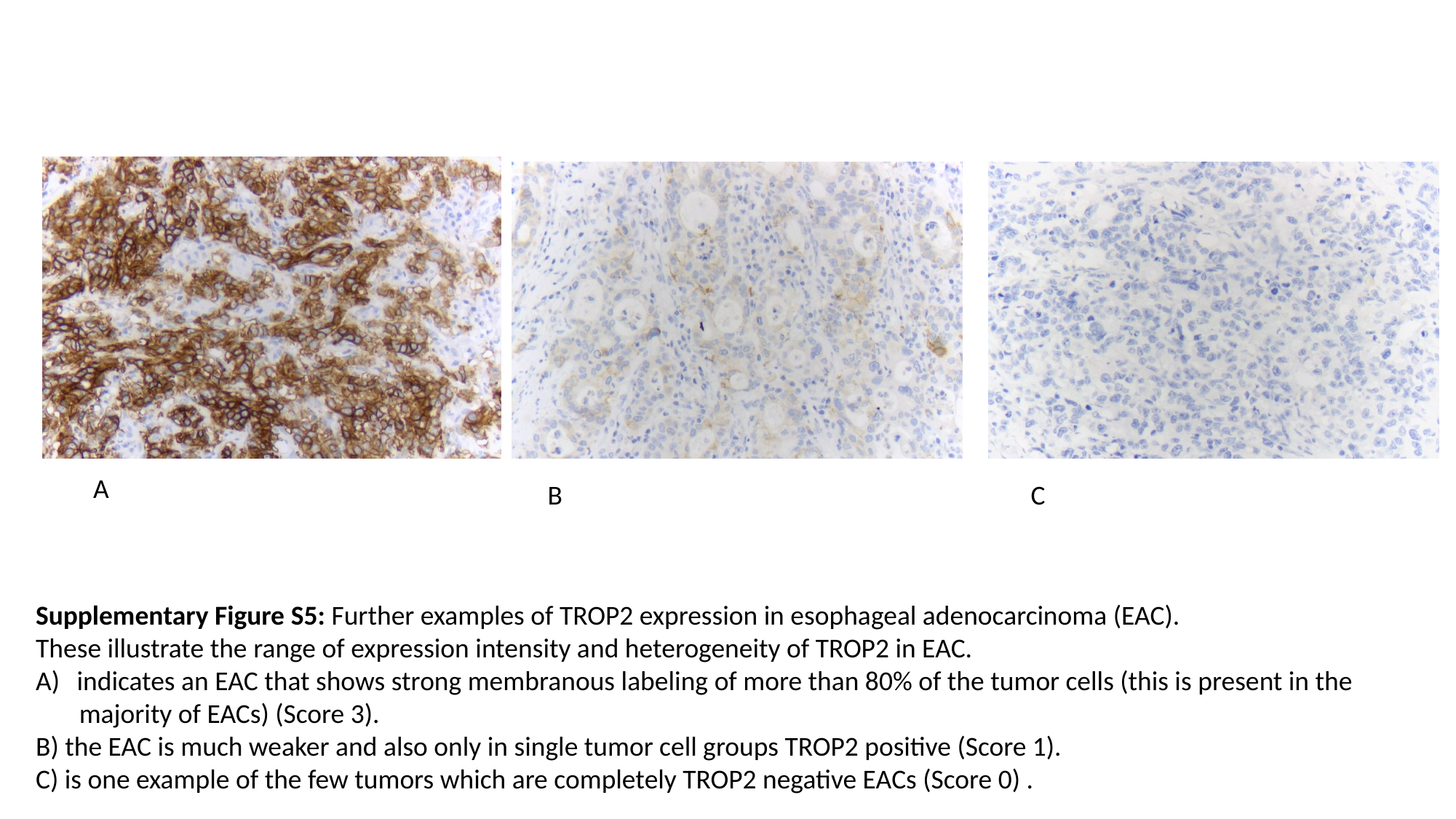

A
B
C
Supplementary Figure S5: Further examples of TROP2 expression in esophageal adenocarcinoma (EAC).
These illustrate the range of expression intensity and heterogeneity of TROP2 in EAC.
indicates an EAC that shows strong membranous labeling of more than 80% of the tumor cells (this is present in the
 majority of EACs) (Score 3).
B) the EAC is much weaker and also only in single tumor cell groups TROP2 positive (Score 1).
C) is one example of the few tumors which are completely TROP2 negative EACs (Score 0) .
